# MISM: A Medical Image Segmentation Metric for Evaluation of Weak Labeled Data

**DOI:** 10.3390/diagnostics13162618

**Published:** 2023-08-08

**Authors:** Dennis Hartmann, Verena Schmid, Philip Meyer, Florian Auer, Iñaki Soto-Rey, Dominik Müller, Frank Kramer

**Affiliations:** 1 IT-Infrastructure for Translational Medical Research, University of Augsburg, 86159 Augsburg, Germany; dennis.hartmann@informatik.uni-augsburg.de (D.H.);; 2 Medical Data Integration Center, Institute for Digital Medicine, University Hospital Augsburg, 86156 Augsburg, Germany

**Keywords:** medical image analysis, biomedical image segmentation, evaluation, performance assessment

## Abstract

Performance measures are an important tool for assessing and comparing different medical image segmentation algorithms. Unfortunately, the current measures have their weaknesses when it comes to assessing certain edge cases. These limitations arise when images with a very small region of interest or without a region of interest at all are assessed. As a solution to these limitations, we propose a new medical image segmentation metric: MISm. This metric is a composition of the Dice similarity coefficient and the weighted specificity. MISm was investigated for definition gaps, an appropriate scoring gradient, and different weighting coefficients used to propose a constant value. Furthermore, an evaluation was performed by comparing the popular metrics in the medical image segmentation and MISm using images of magnet resonance tomography from several fictitious prediction scenarios. Our analysis shows that MISm can be applied in a general way and thus also covers the mentioned edge cases, which are not covered by other metrics, in a reasonable way. In order to allow easy access to MISm and therefore widespread application in the community, as well as reproducibility of experimental results, we included MISm in the publicly available evaluation framework MISeval.

## 1. Introduction

Machine learning algorithms have become increasingly popular in the field of medical image analysis. Neural networks in particular are a driver of this trend due to their state-of-the-art precision [1,2,3]. One area of medical image analysis in which complex problems are investigated is medical image segmentation (MIS). During this process, each pixel of an image is assigned a class, and thus the image is divided into sections, e.g., background, brain, and tumor. In medical imaging, segmentation can be applied, for example, in computer tomography (CT), magnetic resonance imaging (MRI), and optical coherence tomography (OCT). In this field, aspects such as the localization of organs, vessels, and tumors are examined. Because MIS can be integrated into the decision-making process of clinicians, the quality of the predictions is extremely important. In order to quantify the performance, appropriate evaluation measurements are required. However, it has been demonstrated that current measurements have limitations when covering certain edge cases like weak labels [4,5,6,7,8] when a metric is used in isolation. Weak labels, i.e., data with no area of interest in the segmentation mask, are the cases we want to focus on in this paper. These cases are important for evaluation in the medical field, as control patients are common in clinical trials. This weak label data can also be used to verify whether the trained model is prone to misdiagnosis, which can lead to non-acceptance or incorrect treatment of patients in clinical use. However, predictions of weak labels are not taken into account and are rated zero by the dice similarity score, regardless of the correctness of the prediction [9]. It is crucial that control patients’ imaging data are correctly segmented, which is why a metric covering these edge cases is essential.

This is why we present our novel metric MISm, which is a combination of the Dice similarity coefficient and the weighted specificity. Our research aims to develop a MIS metric that overcomes the limitations of current widely used measurements when evaluating weak labeled data. Therefore, the weighted specificity is used when a weak label exists and the Dice similarity score is used in other instances, which results in meaningful output over the entire range of values.

## 2. Materials and Methods

MISm has been included in our previously described package MISeval: a Metric Library for Medical Image Segmentation Evaluation. Throughout this paper, the definition of a metric from Taha et al. is used [6].

### 2.1. Metric Definition

To solve the weak label scoring issue which is present in most metrics in MISm, we propose MISm: Medical Image Segmentation metric

(1) $$MISm=\left\{\begin{array}{cc} DSC=\frac{2TP}{2TP+FP+FN} & \mathrm{if}\;P>0\\ wSpec_{\alpha }=\frac{\alpha *TN}{(1-\alpha )*FP+\alpha *TN} & \mathrm{if}\;P=0 \end{array}\right.$$

The operators are based on the computation of a confusion matrix for binary segmentation, which contains the number of true positive (TP), false positive (FP), true negative (TN), and false negative (FN) predictions. P is the number of the actual positive conditions and therefore the sum of TP and FP. The sum of TN and FN is the number of actual negative conditions; we abbreviate it as N. Furthermore, the weighting coefficient αϵ [0, 1] was introduced.

The first part of our formula is equivalent to the dice similarity or F1-score (DSC). The second part is the Specificity (Spec), also called the True Negative Rate, in which additional weights α and (1 – α) were added. This Spec with added weights, we defined as weighted Specificity (wSpec${}_{\alpha }$). Analogous to the DSC and the Specificity, MISm returns values between [0, 1] with one being equal to the ground truth annotation and zero implying no overlap between ground truth and prediction.

### 2.2. Metric Verification

For the performance measurement of a prediction, common metrics in MIS entail two main limitations. In the presence of weak labels, i.e., when there is no ground truth description, P = 0, and thus TP = FN = 0. TP is 0 because there are no positives and, thus, no true positives. FN is 0 because each pixel or voxel belongs to the background, so this cannot be predicted incorrectly. For this case DSC,

(2) $$DSC=\frac{2TP}{2TP+FP+FN}$$

which is widely used in MIS [2,6,10], takes zero. For FP = 0 or close to zero, the segmentation is an actually accurate true negative, which contradicts the DSC being zero or non-defined. To measure the impact of TN and FP in this case, it is possible to calculate the false positive rate (FPR), also called fall-out, with the formula

(3) $$FPR=\frac{FP}{N}=\frac{FP}{FP+TN}$$

where N is the number of the actual negative condition. If everything is predicted incorrectly, so TN = 0, FPR takes one. To have the common metric range where one means a perfect prediction and zero an incorrect prediction, FPR is reversed and transformed into the Specificity, also called the true negative rate, which is commonly used in medicine.

(4) $$1-FPR=1-\frac{FP}{FP+TN}=\frac{FP+TN}{FP+TN}-\frac{FP}{FP+TN}=\frac{TN}{FP+TN}=Spec$$

The application of the Specificity results in the second limitation for the performance measurement of a prediction: Assume N = 60,000 and P = 0. Let FP = 5000, thus TN = N − FP = 55,000. As almost 10% were predicted to be false positive in this example, we consider a MIS prediction like this to be inaccurate.

(5) $$Spec=\frac{55,000}{5000+55,000}\approx 0.9167$$

To fix this inaccuracy, FP and TN were weighted to each other by adding weights α and 1 – α to the formula. In MIS, according to our experience, far more pixels or voxels are assigned to the background, so TN has a strong influence on Spec, and, according to our experience, often shifts it upwards. Using α as a weighting factor is intended to weaken the weighting of TN and to the same extent using 1−α to strengthen the influence of FP.

(6) $$wSpec_{\alpha }=\frac{\alpha *TN}{(1-\alpha )*FP+\alpha *TN}$$

In the example above, let α = 0.1. This yields to

(7) $$wSpec_{\alpha }=\frac{0.1*55,000}{(1-0.1)5000+0.1*55,000}=0.55,$$

which results in more insightful scoring and represents the second part of the MISm.

MISm takes advantage of wSpec for P = 0, where the DSC is not defined, and the DSC, which is popular in the domain, for all other cases. As a result, MISm is defined for the entire range of values.

### 2.3. Availability

The MISm has been included in MISeval: an open-source Python framework for the evaluation of predictions in the field of medical image segmentation. This framework is available on Github (https://github.com/frankkramer-lab/miseval (accessed on 31 July 2023)).

MISeval is licensed under the open-source GNU General Public License Version 3 (GPL3.0 License), which allows free usage and modification by anyone.

## 3. Results

A theoretical analysis was conducted to examine MISm for definitional gaps and for an appropriate scoring gradient. In addition, different weighting coefficients for MISm were investigated to propose a constant value. To compare MISm with popular metrics, a theoretical application was presented.

### 3.1. Theoretical Analysis

In the following theoretical analysis, definition gaps, as well as an appropriate scoring gradient compared to the DSC and Spec, were investigated. In MIS, FN, FP, TN, and TP are greater than 0 because there is no possibility of a prediction being negative, resulting in the following equation:

(8) TN+FP=0⇔TN=FP=0

The Spec is not defined, if TP ≥ 0, FN ≥ 0, TN = 0 and FP = 0. As P =TP + FN ≥ 0, MISm computes the DSC and is, therefore, defined, although the Spec is not. Analogously, the definition gap of the DSC.

(9) 2TP+FP+FN=0⇔TP=FP=FN=0

Thus, the DSC is not defined for TN ≥ 0 and the other values of zero, which represents a completely true negative prediction. MISm handles this edge case separately by computing the weighted Specificity as P = 0. Therefore, we conclude that our MISm is always defined, in contrast to Spec and DSC.

Analyzing the scoring gradient, we determine that P = 0, but let FP > 0. In this case, DSC = 0 and any prediction will yield the same score, despite a strong possible variation of FP. The fixed scoring outcome is not capable of reflecting the prediction quality properly. In contrast, the Spec grades the predictions, but underweights FP as seen in the example above. As MISm utilizes a weighted Spec in the case P = 0, a more appropriate scoring gradient is sustained.

### 3.2. Weighting Coefficient Analysis

**Figure 1:**
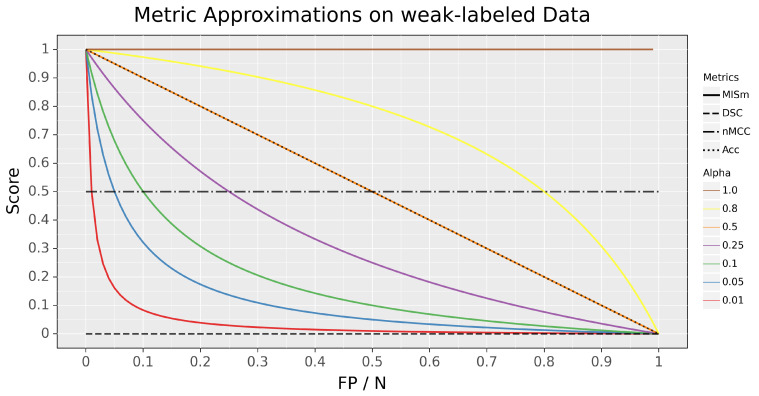
Comparison of the performance metrics considered with the presented MISm in terms of the ratio of false positives to actual negatives if the class observed is not present in the image. The score represents the respective result of each metric for the respective simulation.

Different weighting coefficients were investigated for our metric to determine their impacts on scoring capabilities on the edge case, in which no predictions are present in the mask. To visualize MISm with different weighting coefficients and to compare it with popular metrics of the research field, like the Dice Similarity Coefficient (DSC), normalized Matthews Correlation Coefficient (nMCC), and Accuracy (Acc) [4,6,11,12,13,14], the different scores of these metrics in comparison to the ratio of pixels falsely classified as positive to pixels classified as negative, for the edge case (P = 0), were plotted in Figure 1.

Even though MISm provides a foundation for the evaluation of datasets with control samples, the weighting factor α is a dynamic variable that results in inconsistent performance assessments due to varying weighting factors. The selection of the weighting factor α is still a subjective definition of the assessor, which causes the usage of MISm for quantitative evaluation to be ineffective due to its incomparability as a consequence. In order to utilize MISm as a generic evaluation method for objective performance measurement, it is mandatory to use a fixed and community-accepted weighting factor. The author proposes α = 0.1 as the weighting factor, due to the fact that it represents a reasonable progression for the considered edge case, as shown in Figure 1. Through the progression of the function, the class imbalance often prevalent in the field of MIS is addressed without penalizing small amounts of error too hard. The weighting factor α = 0.1 is implemented in the software MISeval as the default weight for MISm.

### 3.3. Experimental Application

**Figure 2:**
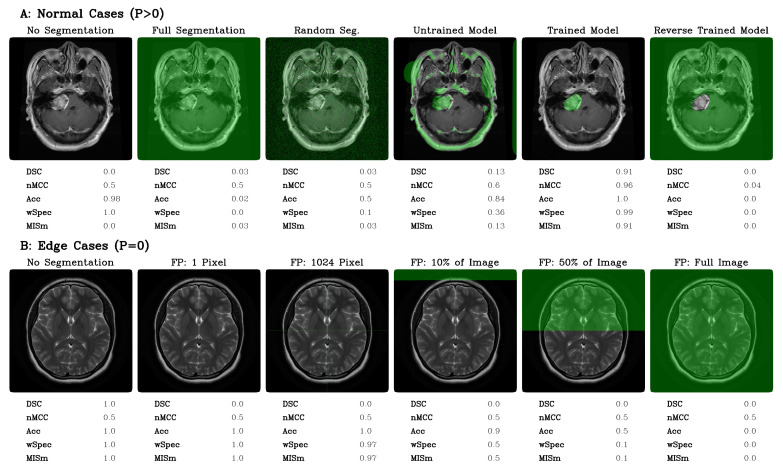
Scoring comparison between MISm and multiple common MIS metrics by application on normal as well as edge cases.

For experimental application, MISm was compared with popular metrics, such as Accuracy (Acc), Dice Similarity Coefficient (DSC) [6], normalized Matthews Correlation Coefficient (nMCC) [4], and weighted Specificity (wSpec), for MRI brain tumor segmentation, which can be seen in Figure 2. The function of these metrics and how they complement each other can be found in the paper by Maier-Hein et al. [15]. The two images in Figure 2 are examples of Cheng et al.’s dataset of T1-weighted CE-MRI scans. Part A shows an MRI scan of the brain with an annotated tumor [16,17]. Based on the annotation, various predicted segmentation cases, in the form of illustrative examples, were tested for evaluation (P > 0). In part B, the edge cases in which no tumor is present in the image (P = 0) were illustrated [18]. For this purpose, segmentations representing the range of MISm for weak labels were chosen as examples. Popular MIS evaluation metrics and the proposed MISm were calculated for the respective cases to allow comparability between them. For wSpec and MISm, α as 0.1 was selected.

## 4. Discussion

MISm proposes a solution to the limitations identified within current gold-standard metrics popular in the field of MIS. By utilizing wSpec, MISm allows the evaluation of datasets with weak label annotations. To guarantee flexibility in the usage, the weighting coefficient α was designed flexibly. However, for performance assessment in the context of evaluation, it is strongly recommended to use the proposed fixed weighting coefficient α = 0.1. In summary, it was shown that MISm is a constantly defined and well-behaving prediction scoring method.

MISm equals DSC if the mask contains an actual positive annotation. As there is no ground truth annotation in the mask, the metrics differ significantly, as shown in our theoretical and experimental application. DSC provides a constant value of zero, whereas MISm has an adequate scoring gradient by decreasing appropriately for each error, starting at a value of one. In comparison to nMCC, which is also widely used in several MIS studies [11,12,13,14], similar limitations as those of DSC were identified, as shown in Figure 1 and Figure 2. Furthermore, the interpretation of nMCC is not always intuitive, because a score equal to zero corresponds to an inversed ground truth annotation, and 0.5 is equivalent to randomness. All other cases of prediction inaccuracy converge to or take a value of 0.5. This is why nMCC insufficiently evaluates the quality of the predictions.

The weighting coefficient analysis and experimental application revealed that the Accuracy is capable of scaling in the absence of actual positives. Still, the score is massively influenced by the inclusion of true negatives due to their significantly higher number in MIS. Even so, the Accuracy is capable of handling our identified edge cases; the true negative inclusion constrains the application in practical settings.

## 5. Future Work

To accelerate the use of MISm in the future, the metric must prove itself in practice and be adapted if necessary. Early experience points to a reduction in α, but no conclusive statement can be made on this yet.

The capabilities of MISm as a loss function for model training are quite promising. Currently, the inclusion of control samples in the training process based on an MIS dataset is rare due to the difficulties of performance assessment for the loss function, as well as the minimal information gain of control samples for the model. However, the significant difficulties that occur when utilizing MIS models in clinical routine [19,20,21,22,23] indicate that current state-of-the-art models from research are often overfitted to their task. Passing images to these models with different medical conditions, from healthy patients or with non-relevant imaging abnormalities like artifacts, drastically reduces performance or leads to complete malfunctioning of the prediction. Training models with loss functions that are also capable of scoring control samples could help reduce the overfitting bias and overcome this challenge. Thus, the integration of the MISm as a loss function into popular neural network frameworks like PyTorch and TensorFlow is planned.

## 6. Conclusions

In this paper, the limitations of several popular MIS metrics were identified and discussed. Whereas our novel metric MISm was proposed, which calculates meaningful values for common as well as edge cases arising in MIS. Unlike the gold-standard DSC or the nMCC, it was proven that even weak labels are evaluated meaningfully with MISm. Furthermore, it was shown that MISm is not as sensitive to a large number of negative pixels in contrast with the Accuracy. In summary, it was demonstrated that MISm can be applied in all possible cases and has appropriate scaling.

MISm was added to MISeval, a publicly available Python framework used to evaluate medical image segmentation: Available online: https://github.com/frankkramer-lab/miseval, (accessed on 31 July 2023).

## Data Availability

Not applicable.

## Abbreviations

The following abbreviations are used in this manuscript:

Acc	Accuracy
CT	Computer tomography
DSC	Dice similarity or F1-score
FN	False negative
FP	False positive
FPR	False positive rate or fall-out
MIS	Medical image segmentation
MRI	Magnetic resonance imaging
N	Negative
nMCC	Normalized Matthews Correlation Coefficient
OCT	Optical coherence tomography
P	Positive
Spec	Specificity or true negative rate
TN	True negative
TP	True positive
wSpec	Weighted specificity
